# Regional blood–brain barrier dysfunction maps to lower performance in cognitive ageing

**DOI:** 10.1093/braincomms/fcag342

**Published:** 2026-10-21

**Authors:** Sheida Mirloo, Sheizaf Shpilberg, Noam Druker, Rotem Saar-Ashkenazy, Ofer Prager, Shai Baruch Neustaedter, Gal Ben-Arie, Ilan Shelef, Lyna Kamintsky, Yonatan Serlin, Danielle K Sandsmark, Alon Friedman, Hadar Shalev

**Affiliations:** Department of Medical Neuroscience and the Brain Repair Center, Dalhousie University, Faculty of Medicine, Halifax, NS B3H 4R2, Canada; Departments of Physiology and Cell Biology, The Zelman Center for Brain Science Research, Ben-Gurion University of the Negev, Beer-Sheva 8410501, Israel; Faculty of Health Sciences, Joyce & Irving Goldman Medical School at Ben-Gurion University of the Negev, Beer-Sheva 8410501, Israel; Departments of Physiology and Cell Biology, The Zelman Center for Brain Science Research, Ben-Gurion University of the Negev, Beer-Sheva 8410501, Israel; Faculty of Social Work, Ashkelon Academic College, Ashkelon 78211, Israel; Departments of Physiology and Cell Biology, The Zelman Center for Brain Science Research, Ben-Gurion University of the Negev, Beer-Sheva 8410501, Israel; Department of Psychiatry, Soroka University Medical Center, Beer-Sheva 8410101, Israel; Department of Diagnostic Imaging, Soroka University Medical Center, Beer Sheva 8410101, Israel; Department of Diagnostic Imaging, Soroka University Medical Center, Beer Sheva 8410101, Israel; Department of Medical Neuroscience and the Brain Repair Center, Dalhousie University, Faculty of Medicine, Halifax, NS B3H 4R2, Canada; Neurophysiology of Epilepsy Unit, NINDS, National Institutes of Health, Bethesda, MD 20892, USA; Department of Neurology, Perelman School of Medicine, University of Pennsylvania, Philadelphia 19104, USA; Department of Medical Neuroscience and the Brain Repair Center, Dalhousie University, Faculty of Medicine, Halifax, NS B3H 4R2, Canada; Center of Neuroscience, The Chaim Sheba Medical Center, Ramat-Gan 5265601, Israel; Department of Psychiatry, Soroka University Medical Center, Beer-Sheva 8410101, Israel

**Keywords:** biomarkers, mild cognitive impairment, MRI, neuropsychological tests

## Abstract

Age-related cognitive decline is a common and growing public health challenge, associated with loss of independence, reduced quality of life, increased caregiver burden and substantial healthcare and societal costs. Yet cognitive decline in ageing is heterogeneous, and current clinical constructs do not reliably capture the underlying biological mechanisms or predict progression to dementia. This cross-sectional study examined whether blood–brain barrier dysfunction (BBBD) provides a biologically supported correlate of cognitive performance beyond clinical diagnosis. The study included 46 patients with mild cognitive impairment (MCI), 46 age-matched controls and 43 young controls. BBBD was assessed using dynamic contrast-enhanced MRI in 42 patients with MCI and all controls, alongside volumetric brain analysis. Standardized NeuroTrax cognitive assessment data were analysed for 41 MCI patients and 19 age-matched controls. BBBD was analysed using the BBBdetect software *(Emagix Inc, Nova Scotia, Canada),* quantifying voxelwise and region-wise blood–brain barrier permeability; volumetric analysis was performed using the Volbrain software; and cognitive performance was stratified into low- and high-performance subgroups based on modified z-scores. Compared with young controls, the older cohort (MCI patients combined with age-matched controls) showed significantly greater BBBD volume and regional extent. Permeability increased with age and showed marked inter-individual variability. Across participants, lower cognitive performance was associated with a greater number of regions exhibiting BBBD. Receiver operating characteristic analysis identified domain-specific spatial patterns of BBBD linked to poor performance in memory, executive, attention, verbal and motor domains, implicating frontal, cingulate, hippocampal, basal ganglia and cerebellar regions. In contrast, volumetric analyses revealed expected age-related reductions in grey and white matter and cortical thickness along with increased ventricular volume; however, these structural changes showed weak to no associations with cognitive performance. These findings suggest that BBBD is widespread in ageing and that regional BBBD maps to domain-specific cognitive deficits. BBBD thus emerges as a biologically relevant biomarker of age-related cognitive impairment and a potential therapeutic target for preserving cognitive function in ageing.

## Teaser

Mirloo *et al.* describe how dynamic contrast-enhanced MRI detects age-related blood–brain barrier dysfunction that is not tied to mild cognitive impairment diagnosis. Regional barrier dysfunction aligns with domain-specific cognitive deficits, whereas volumetric and cortical thickness measures show no such pattern, highlighting it as a sensitive biomarker and therapeutic target.

## Graphical Abstract

**Graphical Abstract:**
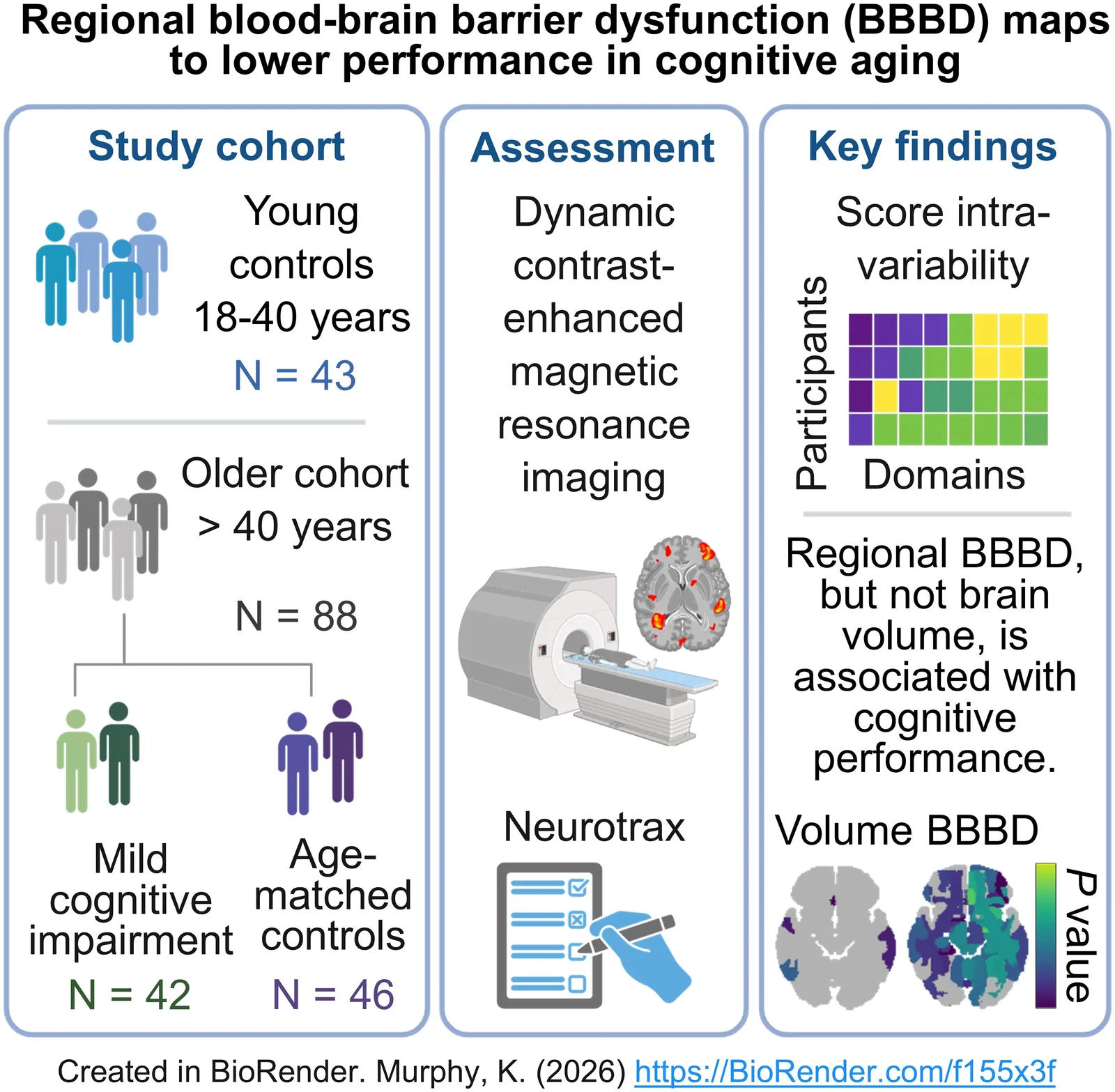

## Introduction

Mild cognitive impairment (MCI) is a clinical entity characterized by cognitive decline that is greater than expected for an individual’s age and education level but does not substantially interfere with activities of daily living.^1,2^ It is often considered an intermediate stage between normal cognitive ageing and dementia, with individuals experiencing noticeable decline in memory, language, problem-solving or judgment. MCI is a heterogeneous syndrome proposed to result from diverse underlying mechanisms, including incipient Alzheimer’s disease, cerebrovascular pathology and other neurodegenerative disorders.^3^ MCI affects 6.7–25.2% of adults over 60, and its prevalence is projected to rise substantially with population ageing by 2050.^4,5^ As a result, MCI represents a major public health challenge, associated with reduced quality of life, neuropsychiatric symptoms and an elevated risk of progression to dementia.^4,6^ However, the diagnosis of MCI remains challenging, and no established biomarkers reliably predict which individuals will progress to dementia or its time course.

The blood–brain barrier (BBB) is a complex structural and functional interface that tightly regulates the brain's environment by controlling the movement of molecules and cells between the bloodstream and neural tissue to maintain homeostasis within the neurovascular unit.^7-9^ Over the past two decades, dynamic contrast-enhanced (DCE) MRI has gained prominence as a preferred method for assessing BBB integrity in living subjects. This minimally invasive technique quantifies BBB permeability by modelling the kinetics of a gadolinium-based contrast agent injected intravenously.^10-13^ The approach involves repeated scanning of the brain following administration of a Gd-based contrast agent to measure changes in magnetic signal due to contrast agent extravasation. DCE-MRI has been successfully used to identify BBB dysfunction (BBBD) in various brain disorders.^14-18^ To date, the application of DCE-MRI in MCI is limited, and the relationship between BBBD and cognitive impairment remains poorly defined. We quantified BBBD with DCE-MRI in patients diagnosed with MCI, age-matched and young healthy participants, and evaluated its association with cognitive performance.

## Materials and methods

### Study design

This prospective observational study included an older cohort of participants aged 40–90 years, comprising patients diagnosed with MCI, recruited from the Memory Clinic at the Department of Psychiatry, Soroka University Medical Center (SUMC) and age-matched volunteers without a formal MCI diagnosis. MCI diagnosis was established by a board-certified psychiatrist also specialized in psychogeriatrics. The diagnosis, based on International Statistical Classification of Disease-10 criteria during standard clinical evaluations, required both a subjective or informant-reported cognitive decline relative to the individual’s prior baseline and a Mini–Mental State Examination score between 21 and 26. This diagnostic approach aligns with the Mild Neurocognitive Disorder frameworks in the Diagnostic and Statistical Manual of Mental Disorders, Fifth Edition and ICD-11, as our study population exhibited objective cognitive impairment without significant loss of functional independence.^19^ Participants without MCI were recruited from among relatives of MCI patients and through advertisements posted on social media.

Forty-six MCI and 19 age-matched healthy individuals underwent standardized NeuroTrax cognitive assessment. Out of 46 MCI, 41 were included in the cognitive assessment analysis; five were excluded because they did not complete all assessment domains. Forty-two MCI patients, 46 age-matched controls and 43 young controls underwent DCE-MRI imaging and were included for BBBD assessment. Medical history was recorded through self-report questionnaires and electronic medical records. MRI results were compared with healthy young controls (18–40 years). Exclusion criteria were contraindications to MRI, contraindications to gadolinium administration (estimated glomerular filtration rate < 60 ml/min), lack of full capacity to provide informed consent or a known history of chronic neurological disorders, including brain tumours, neurodegenerative diseases or significant brain injury.

### Cognitive assessment

Cognitive function was assessed using the validated NeuroTrax computerized battery (www.neurotrax.com), which measures memory, executive function, attention, information processing speed, visuo-spatial abilities, verbal function and motor skills.^20^ NeuroTrax cognitive scores were reported as age- and education-adjusted normalized scores. According to the NeuroTrax normative scoring procedure, raw test outcomes are compared with a normative database of cognitively healthy individuals stratified by age and education. For each outcome, the raw score is converted to a *z*-score using the mean and standard deviation of the appropriate normative stratum and is then transformed to an IQ-style scale, where 100 points represent the normative mean and 15 points correspond to one standard deviation. Thus, scores above or below 100 indicate performance above or below the expected average for an individual’s age and education level, respectively. The global cognitive score was computed as the average of the NeuroTrax domain/index scores. Participants completed the standardized battery on the same day as the MRI scanning, in a quiet environment, following on-screen instructions. Performance scores were automatically adjusted for age and education to generate standardized domain and global cognitive scores.

### Brain imaging

Forty-two MCI patients, 46 older healthy individuals and 43 healthy young participants were scanned on a 3T Philips Ingenia MRI scanner. The image acquisition protocol was performed as previously described.^17,21,22^ Briefly, T_1_- and T_2_-weighted imaging, fluid-attenuated inversion recovery, diffusion- and susceptibility-weighted imaging data were acquired, followed by a DCE sequence. After five dynamic scans, the contrast agent gadoterate meglumine (Gd-DOTA, Dotarem, 0.1 mmol/kg, 0.5 M, 1.5 ml/s) was administered intravenously. Sequences for BBB assessment included anatomical T1W (TR/TE = 8.2/3.7 ms, matrix = 432 × 432, voxel size = 0.5 × 0.5 × 1 mm); axial T_1_-w FFE with variable flip angles (TR/TE = 10/2 ms, matrix = 256 × 256, voxel size = 0.89 × 0.89 × 6 mm, flip angles 10–15–20–25–30°) and dynamic 3D T_1_-w FFE (TR/TE = 4/2 ms, matrix = 192 × 187 reconstructed to 256 × 256, voxel size = 0.9 × 0.9 × 6 mm, flip angle 20°, Δt = 20 s, 60 repetitions).

### Image analysis

BBB permeability analysis was performed as previously reported using the BBBdetect software *(Emagix Inc, NS, Canada).*^22-24^ Briefly, T_1_ intensities were first converted to contrast concentration values,^25^ and concentration-time curves were constructed for each brain voxel. Linear regression was applied to the concentration-time curves between 6 and 20 min post-injection, and the slope of the fitted model was used to estimate the leakage rate Ki (mmol/min).^17,23,26-29^ To account for inter-subject variability in heart rate, blood flow and contrast injection rate, voxelwise leakage rates were normalized to that of the superior sagittal sinus,^17,27,28^ yielding a dimensionless extraction fraction as a measure of leakage rate.

The threshold for BBBD was defined as the median plus two median absolute deviations (MAD) of voxelwise extraction fractions in the young healthy control cohort (*N* = 43). Voxels with leakage rates exceeding this threshold were considered pathological. To assess for region-specific BBBD, each scan was parcellated into 124 anatomically and functionally defined areas in accordance with the Montreal Neurological Institute brain atlas (https://github.com/neurodebian/spm12/tree/master/tpm). For each region, the pathological threshold was similarly defined as the sum of the median plus two MADs of the region extraction fraction in young healthy controls. We then calculated the modified *z*-score for suprathreshold voxels. Regions with a modified *z*-score greater than two MADs were classified as leaky, and the modified *z*-score was further used as an index of leakage extent. For each subject and region, permeability range was defined as the difference between the 90th and 10th percentiles of voxelwise leakage rates. Volumetric, cortical thickness and biological age analyses were performed on T_1_-weighted anatomical scans using the volBrain pipeline.^30^  Δage was defined as chronological age minus MRI-derived biological age; therefore, negative values indicate that biological age exceeds chronological age. Median cortical thickness was calculated as the median value across the frontal, parietal, occipital, temporal, limbic and insular regions.

### Mapping BBBD to cognitive performance

Because cognitive performance and BBBD did not differ significantly between formally diagnosed MCI participants and age-matched controls, we analysed them together as an age-heterogeneous older cohort. This dimensional approach also reduces the impact of potential diagnostic misclassification.

The older cohort was initially stratified into two groups based on the median of the NeuroTrax global cognitive score. As this dichotomization did not yield a significant difference between groups, a more stringent classification was applied using a robust threshold. Specifically, individuals with scores ≥ median + MAD were defined as the high-performance group, and those with scores ≤ median − MAD as the low-performance group. Participants within the intermediate range were not included in group-based comparisons.

To identify brain regions associated with cognitive impairment for each domain, we combined the modified *z*-scored BBBD indices from the low- and high-performance groups into a single vector. We then created a binary label vector, assigning ‘1’ to each observation from the low-performance group and ‘2’ to each observation from the high-performance group. Using MATLAB’s *perfcurve* function with the low-performance group specified as the positive class, we computed receiver operating characteristic (ROC) curves for each region by evaluating true- and false-positive rates across thresholds. From each ROC curve, *perfcurve* returned the area under the curve (AUC), which quantified the ability of regional permeability to discriminate between groups (AUC ≈ 1 indicating strong separation, AUC ≈ 0.5 no discrimination). Regional AUC values were then plotted on brain maps.

### Statistical analysis

Statistical analyses were performed using Prism V.9 (GraphPad Software) and MATLAB. Data are expressed as mean ± standard error of the mean or median (IQR). Normality was assessed with the Shapiro–Wilk test. Group differences were evaluated with two-sided Mann–Whitney U-tests, and associations were examined using Pearson’s correlation coefficient (*r*). For families of related correlation analyses, *P* values were adjusted for multiple comparisons using the Benjamini–Hochberg false discovery rate (FDR) procedure (α = 0.05). Regional analyses involving multiple brain regions were corrected using the Benjamini–Hochberg FDR procedure with *α* = 0.005. Differences between the number of leaky regions in high- and low-performing groups across cognitive domains were assessed using the Wilcoxon signed-rank test.

### Standard protocol approvals, registrations and patient consents

The study was approved by SUMC institutional review board; written informed consent was given by all participants prior to enrolment. The study was conducted in accordance with the Declaration of Helsinki and Good Clinical Practice guidelines. Participant data were anonymized to ensure confidentiality.

## Results

### Participant characteristics

The study included an older cohort with 46 patients recruited from the memory clinic with a diagnosis of MCI (mean age 61.9 ± 1.4 years, 57% female), 46 age-matched controls (mean age 58.4 ± 1.6 years, 62% female) not formally diagnosed and 43 young controls (mean age 27.5 ± 0.6 years, 47% female). Among the 46 patients with MCI, five did not complete all cognitive assessment domains and were therefore excluded from the cognitive analysis; 42 patients underwent DCE-MRI. Two MCI patients lacked a T_1_-weighted image and were therefore excluded from the volumetric, cortical thickness and biological age analyses. Among the 46 age-matched controls, 19 completed NeuroTrax assessments, and all underwent DCE-MRI.

### Cognitive testing

From the older cohort, 41 MCI patients and 19 age-matched controls completed the NeuroTrax cognitive assessment. Across the eight cognitive domains, mean scores were generally centred around 100, except attention and visuo-spatial domains, which showed lower and higher average performance, respectively (Fig. 1A).

**Figure 1:**
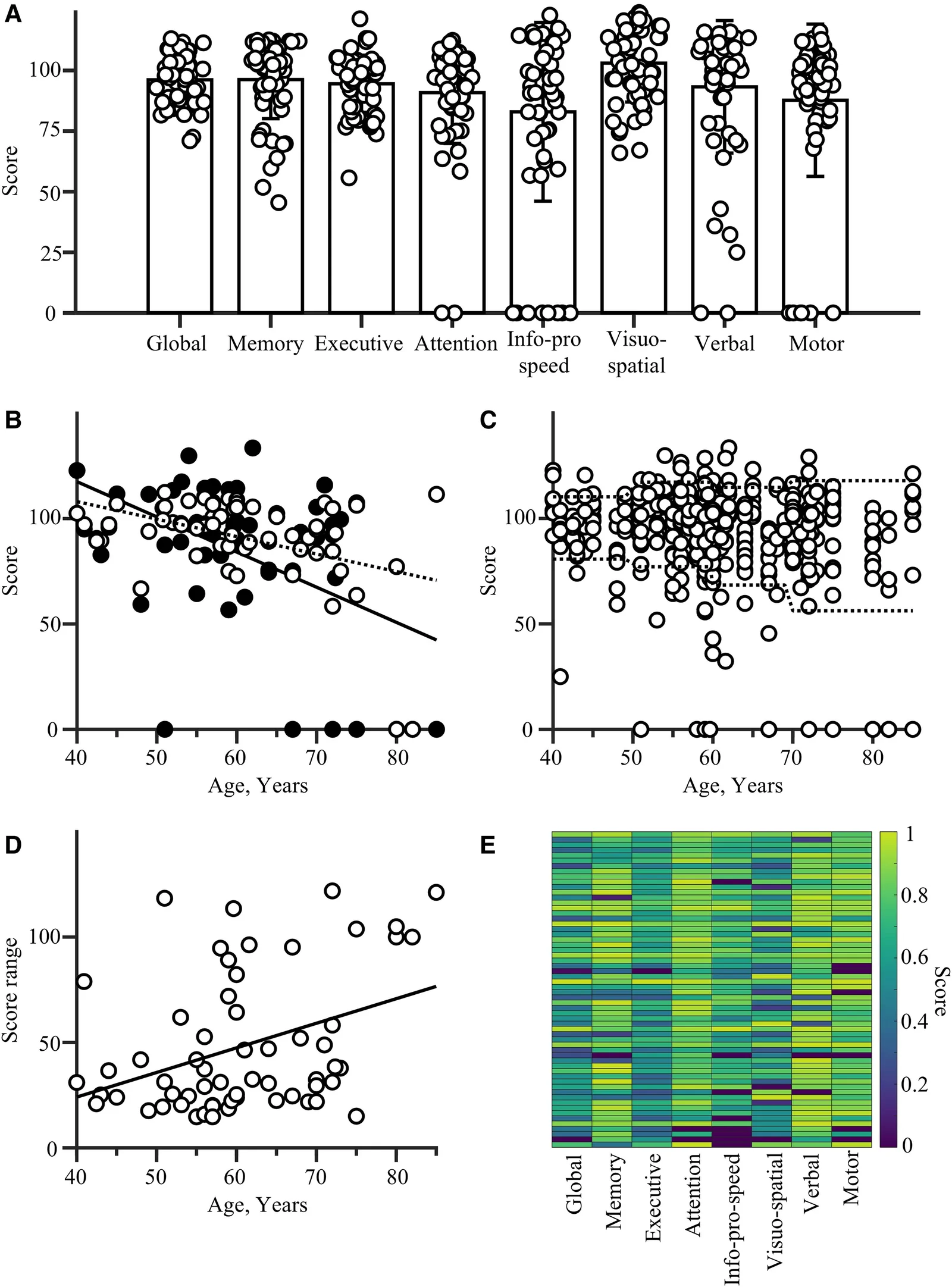
**NeuroTrax cognitive performance.** (**A**) NeuroTrax cognitive scores across eight domains. Bars represent the mean ± standard deviation for each domain, while individual data points indicate participant-level scores. Sample size is consistent across domains (*N* = 60). (**B**) Age-dependent decreases in mean scores were observed only in the Attention domain (empty circles; Pearson correlation, FDR-corrected, *P* = 0.0015, *r* = −0.42, *N* = 60) and Information Processing Speed domain (filled circles; Pearson correlation, FDR-corrected, *P* = 0.0003, *r* = −0.48, *N* = 60). (**C**) Age-dependent increases in variability of NeuroTrax scores are seen in individuals above 40 years old (*N* = 60 per domain). Dashed lines indicate the standard deviation for each age group (40–49, *N* = 8; 50–59, *N* = 23; 60–69, *N* = 14 and ≥70 years, *N* = 15). (**D**) Score range (minimum to maximum) increases with age in individuals over 40 years old (Pearson correlation, FDR-corrected, *P* = 0.0032, *r* = 0.37, *N* = 60). (**E**) Heatmap representing min-max normalized scores across domains, showing individual variability in score and domain (*N* = 60 per domain). FDR, false discovery rate.

An age-related decline was evident in attention and information processing speed (Fig. 1B). Inter-individual variability increased with age: while some older participants maintained high performance across domains, others exhibited pronounced deficits in one or more domains (Fig. 1C and D). No significant differences in cognitive scores were found between participants formally diagnosed with MCI and age-matched controls without a formal diagnosis (Supplementary Fig. 1). Cognitive performance was not uniform within individuals, as participants did not consistently demonstrate either preserved or impaired performance across all domains (Fig. 1E).

### BBB imaging

We next tested whether BBBD was more pronounced in the older cohort compared with young controls. Voxelwise and regional analyses showed individuals with absent, focal or generalized BBBD (see Methods and Fig. 2A). The older cohort showed greater volumes of brain tissue with BBBD (see Methods) than young controls in 120 brain regions out of 124 (Fig. 2B). While no age effect was detected within the young group, the number of regions with compromised BBB increased significantly with age in the older group (*P* = 0.006, *r* = 0.29; Fig. 2C). Region-wise analysis revealed a significant correlation between BBBD and age in 54 brain regions within the older cohort (not corrected for multiple comparisons; Fig. 2D). While no age-related effect on BBBD range (see Methods) was observed in young controls (*P* = 0.14, *r* = −0.23), the older cohort exhibited increased variability in BBBD extent with advancing age (*P* = 0.003, *r* = 0.31, Fig. 2E). Region-wise analysis revealed a significant correlation between BBBD range and age in 43 brain regions in older cohort. These associations were more prominent in the left compared with the right hemisphere (Fig. 2F and Supplementary Table 1).

**Figure 2:**
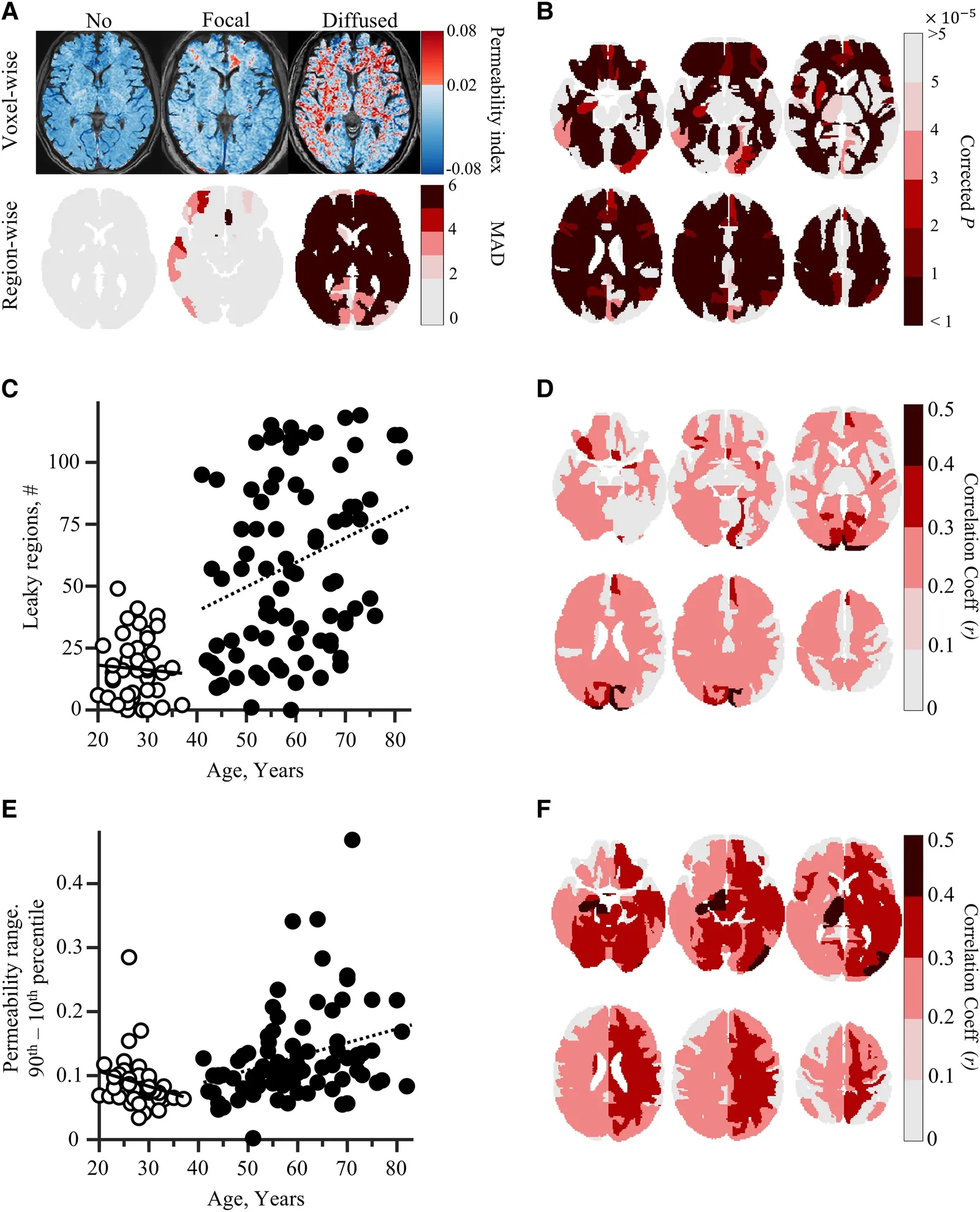
**Age-related blood–brain barrier dysfunction.** (**A**) BBBD maps: Left: healthy young male; middle: focal BBB leakage in a 48-year-old individual; right: diffuse leakage in a 55-year-old individual. Upper panel: voxels with BBBD (>2 MADs above controls) are marked in red. Lower panel: region-wise analysis. Scale bar represents MADs of each region compared with young controls (*N* = 43). (**B**) Statistical brain maps comparing BBBD between older individuals (over-40-year-old participants, *N* = 88) and young controls (*N* = 43). One hundred and twenty out of 124 regions show higher BBBD in older cohort compared to the young controls. Scale bar represents Mann–Whitney U-test *P* values (corrected for multiple comparisons using FDR with α = 0.005). (**C**) Number of leaky regions is age dependent only in older individuals. Young controls (empty circles; Pearson correlation, FDR-corrected, *P* = 0.7, *r* = −0.06, *N* = 43) and older individuals (filled circles; Pearson correlation, FDR-corrected, *P* = 0.012, *r* = 0.29, *N* = 88). (**D**) Brain maps representing Pearson correlation coefficient for age and BBBD for older cohort (*N* = 88). Brain regions with significant correlations (scale bar represents Pearson correlation *P* < 0.05 not corrected for multiple comparisons). (**E**) Whole-brain permeability range (90th–10th percentile of median permeability) is age dependent in older cohort (Pearson correlation, FDR-corrected, *P* = 0.012, *r* = 0.31, *N* = 88) and not in young controls (Pearson correlation, FDR-corrected, *P* = 0.18, *r* = −0.23, *N* = 43). Each data point represents an individual. (**F**) Brain maps showing Pearson correlation coefficient for age and permeability range (10th–90th percentile of median permeability) for older individuals (*N* = 88). Scale bar as in D. BBBD, blood–brain barrier dysfunction; MAD, median absolute deviation; FDR, false discovery rate.

### **Relationship between regional BBBD and cognitive performanc**e

To examine the relationship between regional BBBD and cognitive performance, we analysed all participants from the older cohort, as no differences were found between those who were diagnosed with MCI and age-matched non-diagnosed participants in either BBBD extent (Supplementary Fig. 2) or cognitive performance (Supplementary Fig. 1). For each cognitive domain, participants were stratified into high- and low-performance subgroups (see Methods). Across all cognitive domains, except visuo-spatial function, low-performance participants had more brain regions with BBBD than high-performance counterparts (Fig. 3A and B).

**Figure 3:**
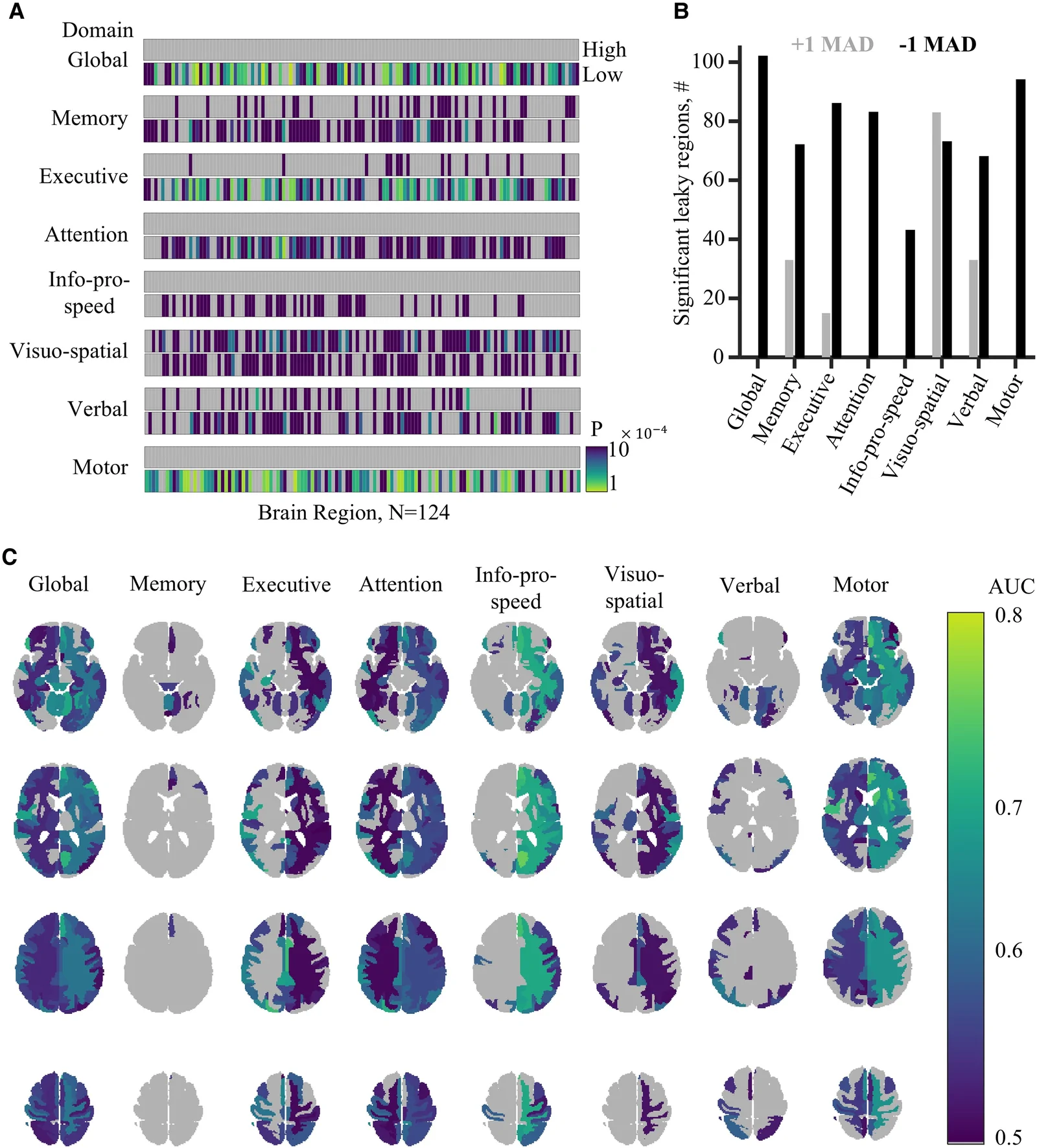
**Blood–brain barrier dysfunction in individuals with cognitive impairment.** (**A**) For each cognitive domain, brain regions (out of 124) showing statistically significant BBBD are marked for participants with high (upper row, > median + MAD) and low (lower row, < median − MAD) cognitive scores. Scale bar represents Mann–Whitney U-test *P* values (corrected for multiple comparisons using false discovery rate with *α* = 0.005). The number of individuals per domain is as follows ($N_{+}$ representing individuals with score > median + MAD, $\;N_{-}$ representing individuals with score < median − MAD): Global ($N_{+}$ = 12, $N_{-}$ = 17), Memory ($N_{+}$ = 9, $N_{-}$ = 21), Executive function ($N_{+}$ = 11, $N_{-}$ = 17), Attention ($N_{+}$ = 13, $N_{-}$ = 15), Information processing speed ($N_{+}$ = 11, $N_{-}$ = 17), Visuo-spatial ($N_{+}$ = 16, $N_{-}$ = 12), Verbal function ($N_{+}$ = 11, $N_{-}$ = 18), Motor skills ($N_{+}$ = 12, $N_{-}$ = 17). (**B)** Bar graph showing the number of brain regions with BBBD in high- (grey bars) versus low-scoring (black bars) individuals (Wilcoxon signed-rank test, one-tailed, *P* = 0.008). Analyses were performed using the same participants as listed for **A**. (**C)** BBBD maps showing regions with AUC > 0.5 in ROC analysis distinguishing between high- and low-performing individuals in each cognitive domain. ROC analyses were performed using the same participants as listed for **A**. BBBD, blood–brain barrier dysfunction; MAD, median absolute deviation; AUC, area under the curve; ROC, receiver operating characteristic.

ROC analysis identified domain-specific BBBD patterns that distinguished low- from high-performing participants. The highest AUC values involved cerebellar, frontal, cingulate, hippocampal, occipital, basal ganglia and insular regions, with distinct spatial patterns across cognitive domains (Fig. 3C; Supplementary Table 2).

### Associations between Δage, BBBD and cognitive performance

In young controls, *Δ*age (see Methods) was close to zero and showed a linear correlation with chronological age (*P* = 0.01, *r* = 0.4). In contrast, no such association was observed in the older cohort (*P* = 0.4, *r* = 0.09; Supplementary Fig. 3A). In older individuals, *Δ*age was larger than in young controls (*P* < 0.00001) and negative (i.e. biological age > chronological age; Supplementary Fig. 3B). No correlation was found between Δage and the number of leaky regions in any group (all *P* > 0.05; Supplementary Fig. 3C), and Δage did not differentiate between low- and high-performance groups across any cognitive domain (Supplementary Fig. 3D–F). Moreover, participants formally diagnosed with MCI and age-matched controls did not differ in Δage (Supplementary Fig. 4).

### Brain volumetry and biological age

We next measured grey matter, white matter and CSF volumes, together with cortical thickness, in the older cohort and young controls. Consistent with previous publications^31,32^ ageing was associated with reduced grey and white matter volumes as well as cortical thickness and higher CSF volume (all *P* < 0.00001, white matter *P* = 0.014; Fig. 4A–D).

**Figure 4:**
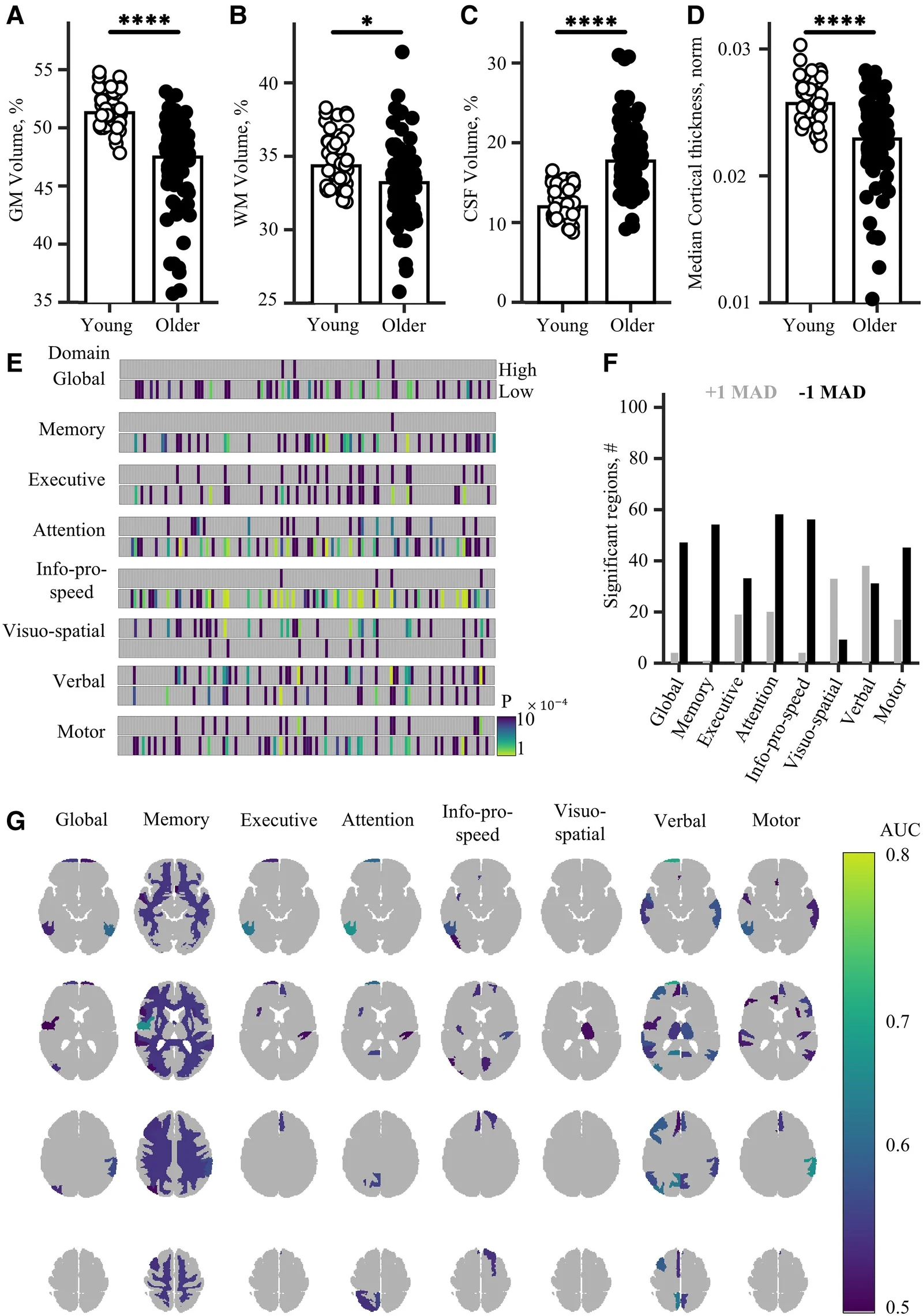
**Brain volumetric analysis.** (**A)** Grey matter (GM), (**B)** white matter (WM), and (**C)** CSF volumes, (**D)** Median cortical thickness in young controls (*N* = 43) and older individuals (*N* = 86). Each data point represents an individual in the corresponding cohort. *: Mann–Whitney U-test *P* < 0.05, ****: Mann-Whitney U-test *P* < 0.00001. (**E**) Brain regions showing statistically significant volume differences between high- (> median + MAD) and low- (< median − MAD) cognitive performance groups compared with young healthy controls, shown for each cognitive domain. Scale bar represents Mann–Whitney U-test *P* values (corrected for multiple comparisons using false discovery rate with *α* = 0.005). The number of individuals per domain is as follows ($N_{+}$ representing individuals with > median + MAD score, $\;N_{-}$ representing individuals with < median − MAD score): Global ($N_{+}$ = 11, $N_{-}$ = 17), Memory ($N_{+}$ = 9, $N_{-}$ = 21), Executive function ($N_{+}$ = 11, $N_{-}$ = 17), Attention ($N_{+}$ = 12, $N_{-}$ = 15), Information processing speed ($N_{+}$ = 10, $N_{-}$ = 17), Visuo-spatial ($N_{+}$ = 16, $N_{-}$ = 12), Verbal function ($N_{+}$ = 11, $N_{-}$ = 18), Motor skills ($N_{+}$ = 11, $N_{-}$ = 17). (**F**) Number of brain regions with lower volume in high- versus low-performing groups across domains (Wilcoxon signed-rank test, *P* = 0.03). Analyses were performed using the same participants as listed for **E**. (**G**) Brain maps showing regions with AUC > 0.5 in ROC analysis distinguishing between high- and low-performing individuals in each cognitive domain. ROC analyses were performed using the same participants as listed for **E**. MAD, median absolute deviation; AUC, area under the curve; ROC, receiver operating characteristic.

Across cognitive domains, a greater number of regions showed reduced volume in the attention, memory and information-processing-speed domains (Fig. 4E). Brain volume showed a difference between high- and low-performing individuals (*P* = 0.02, Fig. 4F); however, this effect was less pronounced compared to BBBD (*P* = 0.006, Fig. 3B). Furthermore, AUC maps did not show distinct regional networks predictive of cognitive performance (Fig. 4G and Table 1).

**Table 1:** AUC and *P* values (low-high cognitive scores, corrected for multiple comparisons) of brain regions volumes in participants with low- compared with high- scores, within the different domains

Modality	Region	*P*-value	AUC
Global	Left inferior temporal gyrus	0.00448	0.61
Memory	Right central operculum	0.00065	0.661
Executive functions	Right inferior temporal gyrus	0.0025	0.631
Attention	Right inferior temporal gyrus	0.00143	0.667
	Right frontal pole	0.00029	0.611
Information processing Speed	-	-	-
Visuo-spatial	-	-	-
Verbal functions	Right frontal pole	0.00485	0.692
	Right precuneus	0.00431	0.631
	Right middle occipital gyrus	0.00485	0.621
Motor	Left supramarginal gyrus	0.00105	0.663

For cortical thickness, the older cohort exhibited lower cortical thickness in 62 brain regions (out of 94 cortical regions) compared with young controls (Supplementary Fig. 5A). A higher number of regions with low cortical thickness was only found in the memory domain in low- compared with high-performing individuals. Overall, no significant differences were found between the high- and low-performance groups. (Supplementary Fig. 5B and C). ROC analysis revealed that within the executive function domain only the medial segments of the precentral gyrus (bilateral) achieved AUC values > 0.6. In the verbal domain, 32 regions demonstrated an AUC ≥ 0.6, with the right planum temporale showing the highest discriminative performance (AUC = 0.79). In the remaining domains, cortical thickness in no region showed good prediction of performance (Supplementary Fig. 5D). Therefore, cortical thickness was not as good as BBBD in distinguishing between low- and high-performance groups. When a similar ROC analysis was performed for *Δ*age, no region demonstrated an AUC ≥ 0.6.

## Discussion

We examined the association between regional BBBD and domain-specific cognitive performance in older adults. Consistent with previous studies, we found age-related increases in both the volume of leaky brain tissue and the number of leaky brain regions. Because BBBD and cognitive performance did not differ significantly between participants with a formal MCI diagnosis and participants not formally diagnosed, we analysed the older participants dimensionally. This approach revealed BBBD network signatures associated with memory, executive, attention, verbal and motor performance, patterns not captured by volumetric or cortical-thickness measures.

Age-related changes in BBB properties have been reported previously in small human cohorts.^33,34^ In recent years, measuring BBB permeability using DCE-MRI has become common, allowing better understanding of cerebrovascular integrity and regional changes in human cohorts. ‘Slow accumulation’ measurements (Veksler model^22^) capture gradual gadolinium accumulation and may be particularly sensitive to low-level transcellular leakage.^22^ Since animal studies indicate increased age-related transcytosis as an underlying BBB permeability increase during ageing,^35-37^ we selected the Veksler model to enhance sensitivity to slow transport. In addition, this model has shown sensitivity for detecting subtle changes in BBB permeability following mild traumatic brain injury^22,38-40^ and transient ischaemic attack.^17^ Moreover, in patients with bipolar disorder^23^ and systemic lupus erythematosus,^24^ this approach was successful in showing that higher permeability is associated with cognitive impairments.

Our observation of BBBD in association with cognitive decline aligns with prior work. In vascular MCI, Li *et al.*^41^ reported higher leakage in cortical grey and white matter; Montagne *et al.*^42^ showed age-related hippocampal leakage with higher permeability in MCI and Vikner *et al.*^43^ reported that for multiple brain regions, BBBD was inversely related to cognition assessed by Montreal Cognitive Assessment (MoCA) score. In a study by Sun *et al.*,^14^ higher K_trans_ values in the hippocampus, thalamus and cingulate cortex were positively correlated with plasma phosphorylated tau 181 levels and negatively correlated with MoCA scores in 18 MCI patients. Extending these findings, our region-wise analysis across 124 areas shows that domain-specific low performance aligns with discrete patterns of BBBD involving frontal, cingulate, hippocampal, basal ganglia and cerebellar structures. This supports a model in which vascular integrity within specific functional networks contributes to selective cognitive vulnerabilities even before overt neurodegeneration.^35^

We observed an age-related increase in inter-individual variability in both the extent of BBBD and cognitive performance, emphasizing the distinction between biological and chronological brain ageing^44^ and highlighting vascular integrity as a candidate marker of biological brain age. Indeed, estimated biological age, derived from anatomical MRI,^30^ was higher than chronological age in >70% of participants included in the older cohort. Importantly, except for the visuo-spatial domain, lower performance across domains was consistently associated with higher regional BBBD, whereas associations with cortical volume and Δage were weaker and not consistently observed (Fig. 3). These results converge with associations reported in non-age-related conditions (e.g. bipolar disorder and systemic lupus erythematosus^23,24^), supporting a cross-diagnostic link between BBBD and cognitive impairments. The distinct spatial patterns we identified for each domain suggest that BBB pathology within specific large-scale networks may contribute to domain-selective deficits.

The mechanisms underlying BBBD-related cognitive impairments are not fully understood. Animal studies demonstrate that experimental BBB opening and extravasation of serum proteins, particularly albumin, activate astrocytes through TGF-β signalling, leading to neuroinflammation, pathological synaptic plasticity and dysfunction of cortical neural networks (for reviews, see^45,46^). Observational human studies have associated BBBD with impaired functional connectivity,^47^ cortical slowing and epilepsy,^48^ linking vascular barrier failure to network-level dysfunction that plausibly degrades cognition. Future human studies should examine whether BBBD co-localizes with changes in electrophysiological markers (e.g. slowing) and functional connectivity within the same individuals.^18^

Reductions in grey and white matter volumes and cortical thickness^49^ and higher CSF volume observed in our study are consistent with prior reports and supports the relevance of our findings to brain ageing. Notably, similar to previous studies, grey-matter loss and ventriculomegaly differentiated MCI from age-matched controls at the group level.^50-53^ However, regional volume, cortical thickness or Δage differences did not map strongly on to domain-specific impairment, suggesting that BBBD may precede, contribute to or operate partly independently of macrostructural atrophy observed in vascular dementia and other neurodegenerative disorders.

Several limitations should be acknowledged. First, the number of age-matched controls who completed cognitive testing was relatively small. Second, the cross-sectional design precludes causal inference; longitudinal studies with repeated cognitive testing and imaging are needed to determine whether BBBD trajectories predict cognitive decline, brain-volume loss or conversion to dementia. Third, unmeasured confounders, including vascular risk factors, medications, sleep and mood disorders and systemic inflammation, may influence both BBB permeability and cognition. Finally, ROC-based AUCs identify discriminative regions but do not establish mechanistic directionality; complementary modelling (e.g. network-level mediation or causal inference) could clarify pathways linking BBBD to behaviour.

In summary, regional BBBD—not a categorical MCI diagnosis—tracked most closely with domain-specific cognitive impairment. These findings motivate translational efforts to (i) validate BBBD-network signatures as prognostic biomarkers, (ii) integrate BBB imaging with plasma markers to improve early detection and (iii) test vascular-protective or BBB-stabilizing interventions with imaging-based endpoints. A better understanding of vascular contributions to age-related cognitive impairment may open new avenues for earlier, more precise detection and for monitoring therapeutic response in trials aimed at preserving or restoring BBB integrity.

## Supplementary Material

fcag342_Supplementary_Data

## Data Availability

Raw data supporting the main and supplementary figures are available upon request. The atlas used for brain region segmentation is available at https://github.com/neurodebian/spm12/tree/master/tpm. No new software or custom algorithms were generated for data analysis for this manuscript. Analysis of blood–brain barrier permeability was performed using the BBBdetect software (Emagix Inc, NS, Canada),^17,22-29^ and analysis of brain volume was performed using the Volbrain pipeline.^30^
