# Supplementary material for: Regional blood–brain barrier dysfunction maps to lower performance in cognitive ageing
Source: Brain Commun. 2026 Oct 21;8(5):fcag342. doi: 10.1093/braincomms/fcag342 (PMC13684410; doi:10.1093/braincomms/fcag342)
Supplement: Supplementary file 1 [file fcag342_supplementary_data.docx]

**Supplementary Figure 1. NeuroTrax cognitive scores in age-matched controls and patients with MCI**. NeuroTrax cognitive scores are shown across eight cognitive domains. Bars represent the mean ± standard deviation for each domain, and individual data points indicate participant-level scores. Sample size is consistent across domains for age-matched controls (empty circles, N = 19) and MCI cohort (filled circles, N = 41). Mann-Whitney U test P values across domains, corrected for multiple comparisons using false discovery rate with α = 0.05, are as follows: Global (*P* = 0.39); Memory (*P* = 0.39), Executive function (*P* = 0.39), Attention (*P* = 0.29), Information processing speed (*P* = 0.5), Visuo-spatial (*P* = 0.39), Verbal function (*P* = 0.52), Motor skills (*P* = 0.39). MCI: Mild cognitive impairment.

**Supplementary Figure 2. BBBD extent in age-matched controls and patients with MCI.** Group comparison of the number of leaky regions in age-matched controls (empty circle, N = 46) and MCI patients (filled circles, N = 42). No significant difference is observed between groups (Mann-Whitney U test, *P* = 0.12). Bars indicate the group medians, with upper error bars representing the bootstrap standard error of the median. Individual data points are overlaid. BBBD: Blood-brain barrier dysfunction; MCI: Mild cognitive impairment.

**Supplementary Figure 3**. $\Delta\boldsymbol{age}$. A. Scatter plot showing the relationship between chronological age and $\Delta\boldsymbol{age}$ (chronological age − biological age) in young controls (Pearson correlation, FDR-corrected, *P* = 0.043, r = 0.4, N = 43) and older individuals (Pearson correlation, FDR-corrected, *P* = 0.52, r = 0.09, N = 86). Young controls cluster tightly around $\Delta\boldsymbol{age}$ ≈ 0, whereas older participants show wider variability, with many individuals exhibiting negative $\Delta\boldsymbol{age}$ values and no clear association with chronological age. Each data point represents an individual participant. B. Group comparisons of $\Delta\boldsymbol{age}$ values in young controls (N = 43) and over-40-year-old participants (N = 86). Young controls show significantly higher $\Delta\boldsymbol{age}$ values compared to over-40-year-old individuals (****: Mann-Whitney U test, *P* < 0.00001). Box plots display the median and interquartile range, with individual data points overlaid. Each data point represents an individual participant. C. Number of leaky regions is not correlated with $\Delta\boldsymbol{age}$ in young and older group. Young controls (empty circles; Pearson correlation, FDR-corrected, *P* = 0.65, r = -0.07, N = 43) and older individuals (filled circles; Pearson correlation, FDR-corrected, *P* = 0.52, r = 0.1, N = 88). Each data point represents an individual participant. D. Brain regions showing statistically significant $\Delta\boldsymbol{age}$ differences between high- (> median + MAD) and low- (< median - MAD) cognitive performance groups compared with young healthy controls, shown for each cognitive domain. The color scale represents Mann-Whitney U test p values (corrected for multiple comparisons using false discovery rate with α = 0.005). The number of individuals per domain are as follows ($N_{+}$ representing individuals with > median + MAD score,$N_{-}$ representing individuals with < median – MAD score): Global ($N_{+}$= 11, $N_{-}$ = 17); Memory ($N_{+}$= 9, $N_{-}$ = 21), Executive function ($N_{+}$= 11, $N_{-}$ = 17), Attention ($N_{+}$= 12, $N_{-}$ = 15), Information processing speed ($N_{+}$= 10, $N_{-}$ = 17), Visuo-spatial ($N_{+}$= 16, $N_{-}$ = 12), Verbal function ($N_{+}$= 11, $N_{-}$ = 18), Motor skills ($N_{+}$= 11, $N_{-}$ = 17). E. Number of brain regions with lower $\Delta\boldsymbol{age}$ in high- versus low-performing groups across domains (Wilcoxon signed-rank test, *P* = 0.31). Analyses were performed using the same participants as listed for panel D. F. Brain maps showing regions with AUC > 0.5 in ROC analysis distinguishing between high- and low-performing individuals in each cognitive domain. ROC analyses were performed using the same participants as listed for panel D. MAD: Median absolute deviation; AUC: Area under curve; ROC: Receiver operating characteristic.

**Supplementary Figure 4.** $\boldsymbol{\Delta}\boldsymbol{age}$ **in age-matched controls and patients with MCI**. Group comparison of the number of $\Delta\boldsymbol{age}$ (chronological age − biological age) in age-matched controls (empty circle, N = 46) and MCI patients (filled circles, N = 40). No significant difference is observed between groups (Mann-Whitney U test, *P* = 0.15). Bars indicate the group median, with individual data points overlaid. MCI: Mild cognitive impairment.

**Supplementary Figure 5. Cortical thickness**. A. Statistical brain maps comparing cortical thickness between older cohort (N = 86) and young controls (N = 43). Color coding for Mann-Whitney U test P values (corrected for multiple comparisons using false discovery rate with α = 0.005); only regions with *P* < 0.005 are color coded. B. Brain regions showing statistically significant cortical thickness differences between high- (> median + MAD) and low- (< median - MAD) cognitive performance groups compared with young healthy controls, shown for each cognitive domain. Color coding for Mann-Whitney U test p values (corrected for multiple comparisons using false discovery rate with α = 0.005); only regions with *P* < 0.005 are color coded. C. Number of brain regions with lower volume in high- versus low-performing groups across domains (Wilcoxon signed-rank test, one-tailed, *P* = 0.85). The number of individuals per domain are as follows ($N_{+}$ representing individuals with > median + MAD score,$N_{-}$ representing individuals with < median – MAD score): Global ($N_{+}$= 11, $N_{-}$ = 17), Memory ($N_{+}$= 9, $N_{-}$ = 21), Executive function ($N_{+}$= 11, $N_{-}$ = 17), Attention ($N_{+}$= 12, $N_{-}$ = 15), Information processing speed ($N_{+}$= 10, $N_{-}$ = 17), Visuo-spatial ($N_{+}$= 16, $N_{-}$ = 12), Verbal function ($N_{+}$= 11, $N_{-}$ = 18), Motor skills ($N_{+}$= 11, $N_{-}$ = 17). D. Brain maps showing regions with AUC > 0.5 in ROC analysis distinguishing between high- and low-performing individuals in each cognitive domain. MAD: Median absolute deviation; AUC: Area under curve; ROC: Receiver operating characteristic.

**Supplementary Tables**

**Supplementary Table 1. Pearson P values (not corrected and corrected for multiple comparisons using false discovery rate with α = 0.05) and correlation coefficient (*r*) for age and permeability range (10th-90th percentile of median permeability) for older individuals (N = 88).**

| **Brain regions** | **P value**  **(not corrected)** | **P value (corrected)** | ***r*** |
| --- | --- | --- | --- |
| Right Hippocampus | < 0.00001 | 0.00331 | 0.42 |
| Right Ventral DC | < 0.00001 | 0.00331 | 0.412 |
| Left inferior occipital gyrus | < 0.00001 | 0.00331 | 0.408 |
| Right Thalamus Proper | 0.00011 | 0.00346 | 0.401 |
| Left posterior insula | 0.00015 | 0.0037 | 0.394 |
| Left calcarine cortex | 0.0003 | 0.00612 | 0.376 |
| Left Ventral DC | 0.00034 | 0.00612 | 0.373 |
| Left anterior insula | 0.00049 | 0.00729 | 0.364 |
| Left fusiform gyrus | 0.00052 | 0.00729 | 0.362 |
| Right lingual gyrus | 0.00065 | 0.00814 | 0.357 |
| Left Thalamus Proper | 0.00077 | 0.00826 | 0.352 |
| Right fusiform gyrus | 0.00079 | 0.00826 | 0.351 |
| Left occipital fusiform gyrus | 0.00099 | 0.00931 | 0.345 |
| Left transverse temporal gyrus | 0.00114 | 0.00931 | 0.341 |
| Left Cerebellum Exterior | 0.00116 | 0.00931 | 0.341 |
| Left medial orbital gyrus | 0.00118 | 0.00931 | 0.34 |
| Left Putamen | 0.00138 | 0.01018 | 0.336 |
| Left Caudate | 0.00151 | 0.01018 | 0.333 |
| Left central operculum | 0.00154 | 0.01018 | 0.333 |
| Right occipital fusiform gyrus | 0.00175 | 0.01073 | 0.329 |
| Right Caudate | 0.00185 | 0.01073 | 0.327 |
| Left Hippocampus | 0.00189 | 0.01073 | 0.327 |
| Right frontal operculum | 0.00196 | 0.01073 | 0.326 |
| Left anterior cingulate gyrus | 0.00211 | 0.01108 | 0.323 |
| Left Cerebral White Matter | 0.0022 | 0.01108 | 0.322 |
| Left planum temporale | 0.00236 | 0.01132 | 0.32 |
| Left superior temporal gyrus | 0.00243 | 0.01132 | 0.319 |
| Right parahippocampal gyrus | 0.00253 | 0.0114 | 0.318 |
| Left planum polare | 0.00301 | 0.01307 | 0.313 |
| Right Amygdala | 0.00318 | 0.01334 | 0.311 |
| Right central operculum | 0.00351 | 0.0134 | 0.308 |
| Left parietal operculum | 0.00357 | 0.0134 | 0.307 |
| Left middle temporal gyrus | 0.00361 | 0.0134 | 0.307 |
| Brain Stem | 0.0037 | 0.0134 | 0.306 |
| Left Accumbens Area | 0.00382 | 0.0134 | 0.305 |
| Cerebellar Vermal Lobules I-V | 0.00383 | 0.0134 | 0.305 |
| Left posterior orbital gyrus | 0.00413 | 0.01373 | 0.303 |
| Right Cerebellum Exterior | 0.00414 | 0.01373 | 0.303 |
| Left precuneus | 0.00444 | 0.01413 | 0.301 |
| Left middle occipital gyrus | 0.00471 | 0.01413 | 0.299 |
| Left lingual gyrus | 0.00481 | 0.01413 | 0.298 |
| Right precuneus | 0.0049 | 0.01413 | 0.297 |
| Left superior occipital gyrus | 0.00491 | 0.01413 | 0.297 |
| Left Cerebellum White Matter | 0.00501 | 0.01413 | 0.297 |
| Left inferior temporal gyrus | 0.00505 | 0.01413 | 0.296 |
| Left medial frontal cortex | 0.00565 | 0.01548 | 0.293 |
| Left cuneus | 0.00604 | 0.01618 | 0.291 |
| Right anterior insula | 0.00657 | 0.01726 | 0.288 |
| Right calcarine cortex | 0.00711 | 0.01829 | 0.285 |
| Right medial frontal cortex | 0.00744 | 0.01844 | 0.284 |
| Right Putamen | 0.00752 | 0.01844 | 0.283 |
| Left precentral gyrus medial segment | 0.00761 | 0.01844 | 0.283 |
| Right Cerebral White Matter | 0.00852 | 0.02024 | 0.279 |
| Left Basal Forebrain | 0.00876 | 0.02043 | 0.278 |
| Right inferior occipital gyrus | 0.00928 | 0.02126 | 0.276 |
| Left occipital pole | 0.00998 | 0.02245 | 0.273 |
| Left frontal operculum | 0.0108 | 0.02331 | 0.271 |
| Left Amygdala | 0.01088 | 0.02331 | 0.27 |
| Right opercular part of the inferior frontal gyrus | 0.01092 | 0.02331 | 0.27 |
| Right parietal operculum | 0.01125 | 0.02362 | 0.269 |
| Right anterior cingulate gyrus | 0.0119 | 0.02457 | 0.267 |
| Right superior occipital gyrus | 0.01274 | 0.02564 | 0.265 |
| Cerebellar Vermal Lobules VIII-X | 0.01282 | 0.02564 | 0.264 |
| Right middle cingulate gyrus | 0.01364 | 0.02672 | 0.262 |
| Left superior frontal gyrus medial segment | 0.01379 | 0.02672 | 0.262 |
| Right middle temporal gyrus | 0.01606 | 0.03067 | 0.256 |
| Right subcallosal area | 0.0185 | 0.0348 | 0.251 |
| Right superior temporal gyrus | 0.01951 | 0.03582 | 0.249 |
| Right inferior temporal gyrus | 0.0197 | 0.03582 | 0.248 |
| Right planum temporale | 0.0199 | 0.03582 | 0.248 |
| Left opercular part of the inferior frontal gyrus | 0.02205 | 0.03882 | 0.244 |
| Right triangular part of the inferior frontal gyrus | 0.02218 | 0.03882 | 0.244 |
| Right Cerebellum White Matter | 0.02278 | 0.03931 | 0.243 |
| Right transverse temporal gyrus | 0.02385 | 0.03982 | 0.241 |
| Left middle cingulate gyrus | 0.02399 | 0.03982 | 0.241 |
| Right postcentral gyrus medial segment | 0.02402 | 0.03982 | 0.24 |
| Left middle frontal gyrus | 0.02885 | 0.04618 | 0.233 |
| Left subcallosal area | 0.02891 | 0.04618 | 0.233 |
| Right angular gyrus | 0.02895 | 0.04618 | 0.233 |
| Cerebellar Vermal Lobules VI-VII | 0.03025 | 0.04765 | 0.231 |
| Right precentral gyrus | 0.0333 | 0.05061 | 0.227 |
| Right orbital part of the inferior frontal gyrus | 0.03342 | 0.05061 | 0.227 |
| Right superior frontal gyrus medial segment | 0.03362 | 0.05061 | 0.227 |
| Left precentral gyrus | 0.03374 | 0.05061 | 0.227 |
| Right cuneus | 0.03544 | 0.05253 | 0.225 |
| Left angular gyrus | 0.03615 | 0.05297 | 0.224 |
| Right posterior insula | 0.03701 | 0.0536 | 0.223 |
| Right Basal Forebrain | 0.03888 | 0.05567 | 0.221 |
| Right supplementary motor cortex | 0.03976 | 0.05629 | 0.22 |
| Right posterior orbital gyrus | 0.04234 | 0.05882 | 0.217 |
| Right entorhinal area | 0.04287 | 0.05882 | 0.216 |
| Right Accumbens Area | 0.04295 | 0.05882 | 0.216 |
| Left posterior cingulate gyrus | 0.0437 | 0.05915 | 0.216 |
| Left superior frontal gyrus | 0.04412 | 0.05915 | 0.215 |
| Left supplementary motor cortex | 0.04558 | 0.06001 | 0.214 |
| Right middle occipital gyrus | 0.04572 | 0.06001 | 0.214 |
| Right precentral gyrus medial segment | 0.04903 | 0.06369 | 0.21 |

**Supplementary Table 2. AUC and P values (low-high cognitive scores, corrected for multiple comparisons) of brain regions with higher BBBD in participants with low- compared with high- scores, within the different domains.**

| **Modality** | **Region** | **P value** | **AUC** |
| --- | --- | --- | --- |
| Global | Cerebellar Vermal Lobules VIII-X; Left calcarine cortex; Left Cerebellum White Matter; Right orbital part of the inferior frontal gyrus; Left triangular part of the inferior frontal gyrus; Left Cerebellum Exterior; Left entorhinal area; Left superior frontal gyrus medial segment; Right central operculum; Left medial frontal cortex; Left fusiform gyrus; Brain Stem; Cerebellar Vermal Lobules VI-VII; Right Cerebellum Exterior; Right Cerebellum White Matter; Right opercular part of the inferior frontal gyrus; Right inferior occipital gyrus; Right superior temporal gyrus; Cerebellar Vermal Lobules I-V; Left anterior cingulate gyrus; Left orbital part of the inferior frontal gyrus; Right parietal operculum; Right Hippocampus; Right superior occipital gyrus; Left Cerebral White Matter; Right Ventral DC; Right postcentral gyrus; Right posterior insula; Left posterior cingulate gyrus; Left superior temporal gyrus; Left anterior insula; Right supramarginal gyrus; Left frontal operculum; Left inferior occipital gyrus; Right fusiform gyrus | 0.00034; 0.00037; 0.00021; 0.0001; 0.00034; 0.00009; 0.00033; 0.00029; 0.00055; 0.00029; 0.00021; 0.00163; 0.00185; 0.00021; 0.00087; 0.0005; 0.00044; 0.00155; 0.00208; 0.00009; 0.00009; 0.00043; 0.0011; 0.00043; 0.00012; 0.00407; 0.0003; 0.00267; 0.00012; 0.00327; 0.00176; 0.00036; 0.00304; 0.0004; 0.00034 | 0.718; 0.711; 0.711; 0.711; 0.706; 0.696; 0.691; 0.691; 0.686; 0.681; 0.681; 0.667; 0.662; 0.657; 0.657; 0.657; 0.652; 0.652; 0.647; 0.647; 0.647; 0.645; 0.635; 0.632; 0.627; 0.627; 0.623; 0.623; 0.618; 0.613; 0.608; 0.608; 0.603; 0.603; 0.603 |
| Memory | Cerebellar Vermal Lobules I-V | 0.0026 | 0.635 |
| Executive Functions | Left middle cingulate gyrus; Right superior occipital gyrus; Right central operculum; Right Hippocampus; Right posterior cingulate gyrus; Left posterior cingulate gyrus; Right superior temporal gyrus; Right inferior occipital gyrus; Left inferior temporal gyrus; Right middle occipital gyrus; Right opercular part of the inferior frontal gyrus; Right supplementary motor cortex; Right supramarginal gyrus; Right middle cingulate gyrus; Left anterior cingulate gyrus; Right orbital part of the inferior frontal gyrus; Left orbital part of the inferior frontal gyrus; Right postcentral gyrus; Right precentral gyrus; Cerebellar Vermal Lobules VIII-X; Right planum polare; Left precentral gyrus medial segment; Left triangular part of the inferior frontal gyrus; Right angular gyrus; Right middle temporal gyrus | 0.00038; 0.00103; 0.00029; 0.00046; 0.00027; 0.00022; 0.00054; 0.00078; 0.00022; 0.00044; 0.00133; 0.00029; 0.00046; 0.00048; 0.00057; 0.00094; 0.00022; 0.00074; 0.00131; 0.00194; 0.0007; 0.00022; 0.00119; 0.00038; 0.00112 | 0.722; 0.722; 0.69; 0.684; 0.684; 0.679; 0.679; 0.668; 0.658; 0.652; 0.647; 0.647; 0.647; 0.636; 0.631; 0.631; 0.628; 0.626; 0.626; 0.615; 0.615; 0.61; 0.61; 0.61; 0.604 |
| Attention | Right superior occipital gyrus; Right inferior occipital gyrus; Left medial frontal cortex; Right occipital pole; Left gyrus rectus; Right supramarginal gyrus; Left Basal Forebrain; Left postcentral gyrus medial segment; Right opercular part of the inferior frontal gyrus; Left calcarine cortex; Right posterior cingulate gyrus; Right middle occipital gyrus; Right superior temporal gyrus; Right medial frontal cortex; Left entorhinal area; Right middle cingulate gyrus; Right orbital part of the inferior frontal gyrus; Left angular gyrus; Left inferior occipital gyrus; Left orbital part of the inferior frontal gyrus; Right cuneus; Left inferior temporal gyrus; Left superior frontal gyrus medial segment; Right supplementary motor cortex; Left occipital fusiform gyrus; Right precentral gyrus medial segment; Right postcentral gyrus; Left anterior insula; Right angular gyrus; Left anterior cingulate gyrus; Left Cerebellum Exterior; Left precuneus; Left superior occipital gyrus; Right central operculum; Left middle cingulate gyrus | 0.00119; 0.0009; 0.00397; 0.00372; 0.00386; 0.00088; 0.00306; 0.00067; 0.00355; 0.00236; 0.00067; 0.0009; 0.00289; 0.00444; 0.00084; 0.00134; 0.00148; 0.0009; 0.00236; 0.00017; 0.00086; 0.00027; 0.0009; 0.00129; 0.00027; 0.0014; 0.00107; 0.0026; 0.00089; 0.00306; 0.00058; 0.0006; 0.00154; 0.0031; 0.0026 | 0.703; 0.682; 0.667; 0.662; 0.659; 0.656; 0.654; 0.651; 0.651; 0.646; 0.646; 0.641; 0.641; 0.636; 0.631; 0.631; 0.631; 0.626; 0.626; 0.626; 0.626; 0.615; 0.615; 0.615; 0.61; 0.61; 0.61; 0.608; 0.605; 0.605; 0.605; 0.605; 0.6; 0.6; 0.6 |
| Information Processing Speed | Left calcarine cortex; Left gyrus rectus; Left superior frontal gyrus medial segment; Left anterior cingulate gyrus; Left medial frontal cortex; Left entorhinal area; Left Cerebral White Matter; Left posterior insula; Left cuneus; Left precuneus; Left transverse temporal gyrus; Right medial frontal cortex; Left Cerebellum Exterior; Left superior temporal gyrus; Right orbital part of the inferior frontal gyrus; Left opercular part of the inferior frontal gyrus; Left anterior insula; Left central operculum; Left planum temporale; Left superior occipital gyrus; Left triangular part of the inferior frontal gyrus; Right inferior occipital gyrus; Left fusiform gyrus; Right cuneus | 0.00349; 0.00361; 0.00193; 0.00173; 0.00161; 0.00161; 0.00161; 0.00399; 0.00287; 0.00231; 0.00378; 0.00308; 0.00161; 0.00373; 0.00287; 0.00161; 0.00373; 0.00428; 0.00378; 0.00287; 0.00274; 0.00287; 0.00308; 0.00161 | 0.751; 0.733; 0.727; 0.717; 0.717; 0.701; 0.695; 0.695; 0.69; 0.679; 0.679; 0.674; 0.674; 0.674; 0.663; 0.658; 0.652; 0.652; 0.642; 0.636; 0.636; 0.62; 0.615; 0.615 |
| Visu-Spatial | Left central operculum; Left inferior temporal gyrus; Left middle temporal gyrus; Left superior temporal gyrus; Right Basal Forebrain; Right Cerebellum White Matter; Left posterior cingulate gyrus; Left middle occipital gyrus; Right central operculum; Left Cerebellum White Matter | 0.00102; 0.00066; 0.00106; 0.00106; 0.00106; 0.00132; 0.00106; 0.0031; 0.00106; 0.00233 | 0.677; 0.677; 0.677; 0.646; 0.646; 0.641; 0.63; 0.609; 0.609; 0.604 |
| Verbal Functions | Right orbital part of the inferior frontal gyrus; Left triangular part of the inferior frontal gyrus; Right angular gyrus; Right superior occipital gyrus; Right middle occipital gyrus; Right Cerebellum Exterior; Right superior parietal lobule | 0.00027; 0.00072; 0.0012; 0.0012; 0.00147; 0.00064; 0.00059 | 0.677; 0.641; 0.636; 0.631; 0.621; 0.606; 0.601 |
| Motor | Left frontal operculum; Left Amygdala; Left anterior cingulate gyrus; Left Caudate; Right central operculum; Left anterior insula; Left Putamen; Left medial frontal cortex; Left entorhinal area; Left fusiform gyrus; Left triangular part of the inferior frontal gyrus; Left calcarine cortex; Left middle cingulate gyrus; Left Cerebral White Matter; Left Hippocampus; Left posterior cingulate gyrus; Left posterior insula; Right opercular part of the inferior frontal gyrus; Right supplementary motor cortex; Right Ventral DC; Left Thalamus Proper; Brain Stem; Left central operculum; Left superior frontal gyrus medial segment; Cerebellar Vermal Lobules I-V; Left Basal Forebrain; Right Thalamus Proper; Left Cerebellum Exterior; Right Hippocampus; Right middle cingulate gyrus; Left Cerebellum White Matter; Left parahippocampal gyrus; Left lingual gyrus; Right posterior cingulate gyrus; Right parietal operculum; Left planum polare; Right superior temporal gyrus; Right Amygdala; Left anterior orbital gyrus | 0.00056; 0.00051; 0.00003; 0.00017; 0.00043; 0.00026; 0.0026; 0.00079; 0.00052; 0.00021; 0.00069; 0.00017; 0.00015; 0.00003; 0.00017; 0.00008; 0.00035; 0.0006; 0.00043; 0.00041; 0.00038; 0.00038; 0.00021; 0.00017; 0.00052; 0.00035; 0.00019; 0.00008; 0.00017; 0.0002; 0.00015; 0.00029; 0.00058; 0.0002; 0.00041; 0.00089; 0.00426; 0.00202; 0.00074 | 0.748; 0.745; 0.74; 0.735; 0.721; 0.713; 0.706; 0.696; 0.691; 0.681; 0.672; 0.672; 0.672; 0.667; 0.662; 0.662; 0.657; 0.657; 0.657; 0.652; 0.652; 0.647; 0.647; 0.647; 0.647; 0.637; 0.632; 0.627; 0.627; 0.627; 0.623; 0.613; 0.613; 0.608; 0.608; 0.605; 0.603; 0.603; 0.6 |
